# DNA Methyltransferases: A Novel Target for Prevention and Therapy

**DOI:** 10.3389/fonc.2014.00080

**Published:** 2014-05-01

**Authors:** Dharmalingam Subramaniam, Ravi Thombre, Animesh Dhar, Shrikant Anant

**Affiliations:** ^1^Department of Molecular and Integrative Physiology, The University of Kansas Medical Center, Kansas City, KS, USA; ^2^The University of Kansas Cancer Center, Kansas City, KS, USA; ^3^Department of Cancer Biology, The University of Kansas Medical Center, Kansas City, KS, USA

**Keywords:** DNMT, colon, pancreas, breast, cancer stem cells, DCLK1

## Abstract

Cancer is the second leading cause of death in US. Despite the emergence of new, targeted agents, and the use of various therapeutic combinations, none of the available treatment options are curative in patients with advanced cancer. Epigenetic alterations are increasingly recognized as valuable targets for the development of cancer therapies. DNA methylation at the 5-position of cytosine, catalyzed by DNA methyltransferases (DNMTs), is the predominant epigenetic modification in mammals. DNMT1, the major enzyme responsible for maintenance of the DNA methylation pattern is located at the replication fork and methylates newly biosynthesized DNA. DNMT2 or TRDMT1, the smallest mammalian DNMT is believed to participate in the recognition of DNA damage, DNA recombination, and mutation repair. It is composed solely of the C-terminal domain, and does not possess the regulatory N-terminal region. The levels of DNMTs, especially those of DNMT3B, DNMT3A, and DNMT3L, are often increased in various cancer tissues and cell lines, which may partially account for the hypermethylation of promoter CpG-rich regions of tumor suppressor genes in a variety of malignancies. Moreover, it has been shown to function in self-renewal and maintenance of colon cancer stem cells and need to be studied in several cancers. Inhibition of DNMTs has demonstrated reduction in tumor formation in part through the increased expression of tumor suppressor genes. Hence, DNMTs can potentially be used as anti-cancer targets. Dietary phytochemicals also inhibit DNMTs and cancer stem cells; this represents a promising approach for the prevention and treatment of many cancers.

## Introduction

DNA methylation and histone modifications are two key players in epigenetic regulation of gene expression in mammalian cells. Epigenetic modifications play an important role in multistage carcinogenesis (1, 2). Extensive analysis of different types of human cancer has revealed that epigenetic alteration of the genome plays a causal role in tumorigenesis. Analysis of epigenetic alterations in tissue samples, together with the histological features of each cancer may aid the understanding of the molecular background of histological heterogeneity in human cancers. DNA methylation, a covalent chemical modification resulting in addition of a methyl group at the carbon 5 position of the cytosine ring in CpG dinucleotides, is one of the most consistent and best known epigenetic events in human cancers. In comparison with normal cells, human cancer cells exhibit global DNA hypomethylation, which can lead to genomic instability, and specific promoter hypermethylation of tumor-suppressor genes, which mediates gene silencing (3). Unlike genetic changes, epigenetic changes can be reversed by pharmacological intervention. Many studies have been focused on understanding the structure and functions of key cellular enzymes that mediate these epigenetic processes and on subsequently developing small molecule inhibitors that target these proteins. In addition, epigenetic alterations are increasingly recognized as valuable targets for the development of cancer therapies.

Methylation of DNA at 5-position of cytosine, catalyzed by DNA methyltransferases (DNMTs), is the predominant epigenetic modification in mammals. Aberrations in methylation play a causal role in a variety of diseases, including cancer. Recent studies have established that like mutation, methylation-mediated gene silencing often leads to tumorigenesis. Paradoxically, genome-wide DNA hypomethylation may also play a causal role in carcinogenesis by inducing chromosomal instability and spurious gene expression. Since methylation does not alter DNA base sequence, much attention has been focused recently on developing small molecule inhibitors of DNMTs that can potentially be used as anti-cancer agents (4). Novel insights provide startling new information regarding DNMTs, with respect to their roles in cancer and the types of proteins they interact with. This information has forced a new view for the role of DNMTs 1, 2, and 3 (5). In this study, we have reviewed the updated information for DNMTs as novel targets for prevention and therapy.

## DNA Methyltransferases

**Figure 1:**
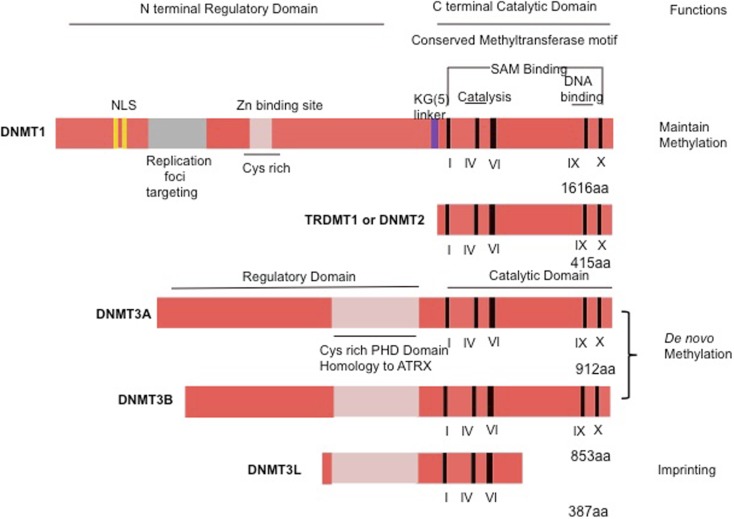
**Schematic representation of the human DNMT1, DNMT2, or TRDMT1 and DNMT3A, 3B, and 3L**. The N-terminal contains motifs of interaction with proteins or DNA. The C-terminal contains the conserved methyltransferases domains. PHD, plant homology domain.

Methylation of mammalian genomic DNA is catalyzed by DNMTs. Patterns of DNA methylation are established by the coordinated action of the DNMTs and associated factors, such as the polycomb proteins in the presence of *S*-adenosyl-methionine that serves as a methyl donor (5). The mammalian DNMT family includes four active members: DNMT1, DNMT3A, DNMT3B, and DNMT3L (6, 7). Mammalian DNMTs are responsible for methylation pattern acquisition during gametogenesis, embryogenesis, and somatic tissue development (5). DNMT1 is the most abundant DNMT involved in the maintenance of methylation (8). DNMT3 functions as a *de novo* methyltransferase and consists of two related proteins encoded by distinct genes, DNMT3A and DNMT3B (9). Of special interest is DNMT2, which has the potential to methylate RNA instead of DNA (10) (Figure 1).

The DNMT1 is the major enzyme responsible for maintenance of the DNA methylation pattern. DNMT1 is also often referred to as maintenance methyltransferase, because it is believed to be the primary enzyme responsible for copying methylation patterns after DNA replication (8). DNMT1 is located at the replication fork and methylates newly biosynthesized DNA (4). The mammalian DNMTs comprised two regions: a C-terminal catalytic portion and a large multi-domain N-terminal region of variable size, which encodes regulatory functions. The C-terminal part is composed of 500 amino acids that are conserved between C5 DNMTs of eukaryotics and prokaryotics, and harbor the active center of the enzyme, containing amino acids motifs characteristic of the cytosine-C5 methyltransferases. The N-terminal region generally contains 621 amino acids that are not essential for DNMT1 activity (4), but are required for discriminating between hemi-methylated and unmethylated DNA. The catalytic domains of all the DNMTs share a common core structure, known as “AdoMet-dependent methyltransferase.” This domain is involved in both cofactor binding (motifs I and X) and substrate catalysis (motifs IV, VI, and VIII). A non-conserved region between motifs VIII and IX, believed to be the target recognition domain, is involved in DNA recognition and specificity (Figure 1). DNMT1 is the most abundant DNMT targeted to replication foci. Three sequences in the N-terminal region increase the precision in maintenance of methylation and give the enzyme direct access to the nuclear replication site: the proliferating cell nuclear antigen (PCNA) binding domain, the replication foci targeting sequence, and the polybromo homology domain. PCNA is required for DNA replication, and the DNMT1–PCNA interaction may allow the newly synthesized daughter strands to be rapidly remethylated before being packaged into chromatin. This tight association of the DNMT1 with the replication machinery allows DNMT1 to bind newly replicated and the naked DNA (11). Without DNMT, some genes may hinder interaction with the replicating foci. Cell-cycle regulator p21 can disrupt DNMT–PCNA interaction, suggesting that p21 may negatively regulate methylation by blocking access of DNMT to PCNA, particularly during DNA damage when p21 protein is induced. Moreover, p21 can itself inhibit DNMT1 gene expression. Under experimental conditions, DNMT1 has up to a 50-fold preference for hemi-methylated DNA substrate and is localized to the replication foci during S-phase. It is proposed to duplicate DNA methylation patterns in the daughter strands during DNA replication (12). Mouse models with both alleles of DNMT1 deleted are embryonic lethal at approximately day E9 (13). The retinoblastoma gene product Rb, another cell-cycle regulator protein, can bind to DNMT1 and inhibit its methyltransferase activity during DNA replication in the cell cycle. Loss of functional Rb may grant DNMT1 free access to the genome, which could allow aberrant *de novo* methylation of CpG. These observations point to a complicated network of connections between DNMT1 and several cellular proteins involved in gene regulation and epigenetic signaling during cell replication (4).

DNMT2 is the smallest mammalian DNMT and it is now termed as TRDMT1. It is composed solely of the C-terminal domain, and does not possess the regulatory N-terminal region. The structure of DNMT2 suggests that this enzyme participates in the recognition of DNA damage, DNA recombination, and mutation repair (14). DNMT2 is a methyltransferase homolog that methylates cytosine-38 in the anticodon loop of aspartic acid transfer RNA instead of DNA (15).

DNMT3A and DNMT3B cannot differentiate between unmethylated and hemi-methylated CpG sites, and they cannot copy a specific pattern of methylation or contribute to the maintenance of methylation pattern (16). Because they show no preference for hemi-methylated DNA, both enzymes appear to function as *de novo* methyltransferases (16) and show a disperse distribution throughout the nucleus not associated with replication sites, even during S-phase (17). This finding suggests that these DNMTs utilize a different mechanism for accessing the densely packed chromatin and for interacting with their target sites that may involve auxiliary factors such as chromatin remodeling complexes (18). Mice lacking DNMT3A die at about 4 weeks of age, whereas DNMT3B knockout induces embryonic lethality at E14.5–E18.5 (16). Possessing homology to DNMT3A and DNMT3B, DNMT3L assists the *de novo* methyltransferases by increasing their ability to bind to the methyl group donor, *S*-adenosyl-*L*-methionine, and stimulating their activity *in vivo* (19), although DNMT3L has no catalytic activity itself. DNMT3L homozygous-null mice are viable, whereas heterozygous embryos derived from homozygous DNMT3L-null oocytes die around E9 and display impaired maternal methylation imprints and biallelic expression of imprinted genes normally expressed only from the allele of paternal origin (20). Cooperation among different DNMTs is also required in methylating some regions of the genome, particularly repetitive elements. It is widely speculated that DNMT1 acts in “maintenance” of methyltransferase during DNA synthesis and that DNMT3A and DNMT3B act as “*de novo*” enzymes in development. However, mounting evidence indicates that DNMT1 may also be required for *de novo* methylation of genomic DNA and that DNMT3A and DNMT3B contribute to maintain methylation during replication (21).

DNMT3A and DNMT3B are highly expressed in early embryonic cells, the stage in which most programed *de novo* methylation events occur, are downregulated after differentiation and in adult somatic tissues, and are overexpressed in tumor cells (22). DNMT3B has been shown to play a crucial role in incorporating *de novo* hypermethylation of promoter CpG islands, a possible mechanism for tumor suppressor gene inactivation within human cancer cells (22). Another member of the DNMT3 family is DNMT3L, a regulatory factor for *de novo* methylation without methylation capacities (23). Its amino acid sequence is very similar to that of DNMT3A and DNMT3B but lacks the residues required for DNMT activity in the C-terminal domain (23). Additional studies are required for the precise role for DNMT3L.

Recent studies suggest that an interaction between DNMT1 and DNMT3B may be vital for the maintenance of patterns of DNA methylation in human colon-cancer cells, particularly in repeat regions and imprinted genes (23). During early embryogenesis, *de novo* DNA methylation is mediated by DNMT3A and DNMT3B associated with DNMT3L. It has recently been reported that in the cell, DNMT3A and DNMT3B are tightly associated with nucleosomes containing methylated DNA (23). Both the direct interaction of these proteins with the histone tails and the polymerization of DNMT3A could contribute to the stable association of these enzymes with chromatin. The levels of DNMTs, especially those of DNMT3A and DNMT3B, are often increased in various cancer tissues and cell lines. This may partially account for the hypermethylation of promoter CpG-rich regions of tumor suppressor genes in a variety of malignancies (24).

## DNA Methyltransferases Overexpression and Cancer

The human DNMTs 1, 3A, and 3B coordinate mRNA expression in normal tissues and overexpression in tumors (25). The expression levels of these DNMTs are reportedly elevated in cancers of the colon (26), prostate (27), breast (28, 29), liver (30), and in leukemia (31). The role of altered expression of DNMTs in DNA hypomethylation and hypermethylation in cancer is uncertain and may involve changes in mRNA or protein expression. There is considerable evidence indicating an up-regulation of DNMT1 in cancer (32, 33). In addition, experimental evidence indicates that forced overexpression of the murine DNMT1 gene in NIH3T3 cells results in cellular transformation (34). In human fibroblasts, sustained overexpression of DNMT1 leads to the processive time-dependent hypermethylation of a number of CpG islands (35). DNMT1 expression is upregulated following fos overexpression and appears to play a role in the fos-induced cell transformation (36). Conversely, reduction of DNMT1 levels appears to have protective effects. Mice predisposed to colonic polyp formation (Min mice) develop fewer polyps in a DNMT1 heterozygous background. Similar results are seen when treated with 5aza-dC (37). Reduction of DNMT1 through an antisense approach also blocks tumorigenesis (38–41). Interestingly, deletion of the DNMT1 gene in a colon cancer cell line (HCT116), while resulting in slower growth, diminished genomic methylation levels modestly ~20% (42). In particular, aberrant CpG island methylation was retained suggesting that another methyltransferase maintains this methylation. Like DNMT1, the aforementioned DNMT3A and DNMT3B enzymes also appear to be modestly overexpressed in cancer. Therefore, the balance of all three enzymes and their accumulative and coordinated effects must be studied (25, 43). DNMTs and its importance in specific cancers are summarized in Table 1.

**Table 1:** **DNA methyltransferases and its importance in specific cancer**.

DNA methyltransferases	Importance in specific cancer	Reference
DNMT1	Leukemia: upregulated – 5.3-fold expression	Mizuno et al. (44)
	Gastric cancer – 64.8% localized in the cytoplasm and nuclei	Yang et al. (45)
	Breast cancer – 16.6%	Girault et al. (28)
	Hepatocellular carcinoma – 100%	Nagai et al. (46)
	Pancreatic cancer – highly expressed – Gli target gene	He et al. (47)
	Colon cancer – highly expressed	Robertson et al. (25)
	Glioblastoma – overexpressed	Rajendran et al. (48)
DNMT2 or TRDM T1	Hepatocellular carcinoma – reduced expression	Saito et al. (49)
	Colorectal and stomach cancers – lower mRNA expression	Kanai et al. (50)
DNMT3A	Acute myeloid leukemia – 22.1% mutations and affect translation	Ley et al. (51)
	Gastric cancer – 70.4% localized in the cytoplasm	Yang et al. (45)
	Breast cancer – 14%	Girault et al. (28)
	Hepatocellular carcinoma – 60%	Nagai et al. (46)
	Pancreatic cancer – highly expressed – regulated by Gli1	He et al. (47)
	Colon cancer – highly expressed	Robertson et al. (25)
DNMT3B	Leukemia: upregulated – 11.7-fold expression	Mizuno et al. (44)
	Gastric cancer – 51.9% localized in the cytoplasm	Yang et al. (45)
	Breast cancer – 81.8% poor prognosis	Girault et al. (28)
	Breast cancer cell lines-hypermethylation defect resulted in aberrant – overexpression DNMT activity	Roll et al. (52)
	Hepatocellular carcinoma (60%) and mRNA levels high	Nagai et al. (46)
	Colon cancer – highly expressed	Robertson et al. (25), Ibrahim et al. (53)
	Prostate cancer – overexpressed	Kobayashi et al. (54)
	Glioblastoma – overexpressed	Rajendran et al. (48)
DNMT3L	Cervical cancer – promising biomarker	Gokul et al. (55)
	Embryonal carcinoma – novel biomarker	Minami et al. (56)

## Colon Cancer

Colorectal cancer is the second leading cause of death in the United States and is a major health problem globally (57). The lifetime risk of developing colorectal cancer in both men and women is about 1 in 20 (5.1%) (58). Colorectal cancer affects over 146,970 individuals yearly, and accounts for around 49,920 deaths (59). The American Cancer Society (ACS) estimated 96,830 new cases (48,450 men and 48,380 women) would be diagnosed with colon cancer during 2014 and also estimated 50,310 deaths (26,270 men and 24,040 women) (60–62). Screening for colon cancer can be done by colonoscopy to find polyps, and removing these polyps at an early stage can prevent cancer progression. When the polyps are allowed to persist in the colon for a long time, they may develop into cancer. Hence, regular colonoscopy is recommended in the United States for those over 50 years of age (63). Recurrence of colon cancer is common, with an estimated 40% of cases returning within 3–5 years of treatment. Chemotherapeutic compounds currently being used for the treatment of colorectal cancer include 5-fluorouracil, Oxaliplatin, and Irinotecan hydrochloride or drug combinations FOLFOX or FOLFIRI. Because conventional therapies, including surgical resection, chemotherapy, and radiation are often inadequate in treating this disease, new treatment options are critically needed. Despite the emergence of novel targeted agents and the use of various therapeutic combinations, no treatment options are available that are curative in patients with advanced cancer. More recently, the cancer stem-cell concept is gaining importance, because it suggests new approaches to anti-cancer therapies (64, 65). Cancer tissues are composed from several heterogeneous cancer cells and a small population of cancer cells is supposed to have higher tumor-initiating ability. These higher tumorigenic populations are named “cancer stem cells (CSCs)” or “cancer initiating cells (CICs).” CSCs/CICs are defined as small population of cancer cells which has (1) higher tumor-initiating ability, (2) self-renewal, and (3) differentiation (66–71). CSCs/CICs have been reported to be resistant to chemotherapy, radiotherapy, and certain molecular targeting therapies (72); thus, elucidation of the molecular mechanisms of the maintenance of CSCs/CICs should be useful for establishing efficient CSC/CIC targeting treatment. Potential markers of colorectal cancer stem cells have been proposed, including CD133, CD166, CD24, CD44, ALDH1, LGR5, and DCLK1 (65, 73–76). Most recently, Nakanishi and colleagues demonstrated that doublecortin and CaM kinase-like-1 (DCLK1) distinguishes between tumor and normal stem cells in the intestine. Their studies demonstrated that specific ablation of DCLK1^+^ stem cells resulted in a marked regression of polyps without apparent damage to the normal intestine and could be a therapeutic target for colon cancer (77, 78).

### Colon cancer and DNMTs

DNMT1 and DNMT3B modulate distinct polycomb-mediated histone modifications in colon cancer (79). Manipulation of DNMT1 levels has been used as a tool to study the effect of DNA hypomethylation on tumorigenesis in several *in vivo* studies. The role of DNA methylation *in vivo* was first explored in the intestine using the *APC^Min/^*^+^, a commonly used mouse model of intestinal tumors since it very closely mimics the human familial adenomatous polyposis (FAP) condition (80–82). These mice develop benign intestinal tumors with a rare occurrence of malignant cancers (81). Treatment of *APC^Min/^*^+^ mice with the demethylating agent, 5-aza-2′-deoxycytidine, significantly reduces tumor formation in the intestine, suggesting that DNA methylation may play an important role during tumorigenesis (80). Moreover, overexpression of DNMT3B1 in the *APC^Min/^*^+^ model enhanced colorectal carcinogenesis and caused tumor suppressor gene methylation (83). Other studies have also shown that crossing *APC^Min/^*^+^ mice with DNMT1 hypomorphic mice results in complete suppression of macroscopic intestinal neoplasia (84). Reduced DNMT1 expression also affects the frequency of malignant intestinal tumors in DNA mismatch repair deficient mice (*Mlh1*^−/−^) (85, 86).

### Colon cancer stem cells and DNMTs

Previous studies have reported that hematopoietic stem cell self-renewal can be abrogated by conditional gene knockout of DNMT1, while the mature differentiated hematopoietic lineage is not affected (87). DNMT1 is essential for maintenance of the leukemia stem cells of bilinear myeloid-B lymphoid leukemia induced by transduction of c-Myc and Bcl-2 (88). DNMT1 was also shown to be essential for the self-renewal of skin progenitor cells (89). Most recently, studies have demonstrated that DNMT1 functions in the maintenance of human colon CSCs/CICs using the human colon cancer cell line HCT116 and its DNMT1 somatic knockout variant (*DNMT1*^−/−^). The rates of CSCs/CICs were evaluated by side population (SP) analysis, ALDEFLUOR assay, and expression of CD44 and CD24. SP, ALDEFLUOR^+^ and CD44^+^ and CD24^+^ (CD44^+^CD24^+^) cell rates were lower in *DNMT1*^−/−^ cells than in control HCT116 cells. Since CSCs/CICs have higher tumor-initiating ability than that of non-CSCs/CICs, the tumor-initiating ability was also addressed by injecting immune deficient NOD/SCID mice (90). *DNMT1*^−/−^ cells showed less tumor-initiating ability than did control HCT116 cells, whereas the growing rate of *DNMT1*^−/−^ cells showed no significant difference from that of HCT116 cells both *in vitro* and *in vivo* (90). Similar results were obtained for cells in which DNMT1 had been transiently knocked-down using gene-specific siRNAs. These results indicate that DNMT1 is essential for maintenance of colon CSCs/CICs and that short-term suppression of DNMT1 might be sufficient to disrupt CSCs/CICs (90).

## Pancreatic Cancer

Pancreatic cancer is an aggressive malignancy with one of the highest mortalities among all cancers. It is the fourth leading cause of cancer death in the United States with <5% 5-year survival rate. The lifetime risk of developing pancreatic cancer in both men and women is about 1 in 79 (1.27%) (91). The ACS estimated that new cases of 46,420 Americans (23,530 men and 22,890 women) would be diagnosed with pancreatic cancer during 2014. The ACS also estimated that 39,590 Americans (20,170 men and 19,420 women) would die of pancreatic cancer in 2014 (60, 62). Despite advances in molecular pathogenesis, problems such as drug resistance and susceptibility for metastasis make pancreatic cancer a major unsolved health problem in the United States (92). Unfortunately, pancreatic cancer is a rapidly invasive, metastatic tumor that is resistant to standard therapies (93). At present, single agent-based chemotherapy (e.g., gemcitabine) is the mainstay treatment for metastatic pancreatic adenocarcinoma. Recent data indicate that in addition to Gemcitabine and a 5-FU plus, a platinum agent such as Oxaliplatin could be used as a therapeutic paradigm for early-stage cancer patients (94). However, none of the available current chemotherapeutic agents have objective response rates of over 10% (95, 96). The magnitude of this problem mandates the need for novel therapeutic agents. Recently, CSCs and epithelial–mesenchymal transition (EMT)-type cells, which share molecular characteristics with CSCs, have been postulated to play critical roles in drug resistance and cancer metastasis in pancreatic cancer (97). Recent studies suggest that CD44^+^CD24^+^ESA^+^ (epithelial specific antigen) and ALDH1 could potentially be pancreatic cancer stem-cell markers (98, 99). In addition, we have determined that an identified intestinal stem-cell marker DCLK1 is also expressed in a small proportion of cells in the pancreas and in pancreatic cancer stem-cell marker (100, 101).

### Pancreatic cancer and DNMTs

Aberrant DNA hypermethylation patterns have been observed in both early- and late-stage human pancreatic tumors (86, 102). Oghamian et al. hypothesized that reduction in DNA methylation levels may decrease pancreatic tumor burden *in vivo* (86). Mice heterozygous for mutation of APC gene are predisposed to the development of benign intestinal polyps, whereas mice homozygous for a mutation in the *Trp53* gene develop a wide range of malignancies, including sarcomas and lymphomas (103). The combined mutation of *APC^Min/^*^+^and *Trp53*^−/−^ has been shown to result in a shift in phenotype with nearly 83% of the animals developing abnormalities of the exocrine pancreas, of which 22% also possessed pancreatic acinar cell carcinoma (104, 105). Oghamian et al. also studied the role of DNA methylation in pancreatic tumorigenesis, using this *APC^Min/^*^+^*Trp53*^−/−^mouse model of exocrine pancreatic cancer and crossed it with mice carrying hypomorphic alleles of DNMT1. They found that tumor burden, but not tumor size, is significantly reduced with decreasing DNMT1 levels, suggesting that DNA methylation is involved in pancreatic tumorigenesis in this mouse model. Their detailed analyses also showed that the reduction in tumor burden is the result of a decrease in both early- and late-stage lesions. In addition, they observed decreased levels of DNA methylation at candidate genes in the normal pancreas of DNMT1 hypomorphic mice (86). Moreover, the expression of DNMT1 protein increased with the development of pancreatic cancer from normal tissue to precancerous lesions (PanINs) and to cancer (PDAC) (106–109). Clinic pathological analyses by Wang and colleagues suggested that PDAC patients with higher DNMT1 protein expression had an overall lower survival rate than those with lower expression. Moreover, higher DNMT1 expression correlated with advanced stages of the disease, reflecting the malignancy potential of PDAC (109). Recent studies examining DNMT mRNA expression in pancreatic cancer has also demonstrated that the levels of the three DNMTs increased with the development of pancreatic cancer from normal duct to pancreatic intraductal neoplasia and further to PDAC. In a statistical study with TNM staging and history of chronic pancreatitis, DNMT3A and DNMT3B, but not DNMT1 expression, correlated with tumor size. Patients with higher levels of DNMT3A, and/or DNMT3B expression had an overall lower survival than those with lower levels of expression (110). Furthermore, univariate analysis showed that high expression levels of DNMTs, in concert with alcohol consumption, tumor differentiation, and TNM staging were statistically significant risk factors. Multivariate analyses showed that high level of DNMT3B expression and tumor differentiation were statistically significant independent poor prognostic factors. Their results suggested that pancreatic carcinogenesis involves an increased mRNA expression of three DNMTs, and they may become valuable diagnostic and prognostic markers as well as potential therapeutic targets for pancreatic cancer (110). Moreover, immunohistochemistry result suggested the expressions of GLI1, DNMT1, and DNMT3a in pancreatic cancer tissues were higher than those in adjacent normal tissues. DNMT1 and DNMT3a are regulated by GLI1 in pancreatic cancer (47).

## Breast Cancer

Breast cancer is the most common form of cancer diagnosed in women worldwide, accounting for 23% (1.38 million) of the total new cancer cases and 14% of the cancer deaths in 2008 (111, 112). Although the rate of mortality as a result of breast cancer has decreased in western countries, including the USA, in part due to early detection. ACS estimates that 235,030 new breast cancer cases will be identified in the United States in 2014 with an estimated death rate of 40,430 (15%) (60, 62). Breast cancer has a heterogeneous predisposition at both the histological and molecular levels. At least six distinct subtypes have been described on the basis of gene expression profiling, with the most important determinants of these subtypes being the presence or absence of expression of the estrogen or progesterone receptor or the amplification/overexpression of the HER2/ERBB2 locus (113). Subgroups of breast cancer are frequently distinguished into luminal A (estrogen/progesterone-positive), luminal B, HER2^+^, and so-called “triple negative” subtypes (114). Despite the ability of these subtypes to predict outcome, patient response to chemotherapy or targeted therapy remains variable. The current standard of therapy for breast cancer includes surgical resection, radiation, and chemotherapeutic agents such as cisplatin, pacliataxel, carboplatin, bevacizumab, doxorubicin, cyclophosphamide, docetaxel, and epirubicin (115).

Our understanding of CSCs comes primarily from studies on breast cancer stem cells (BrCSC). These have been isolated from human breast tumors or breast cancer-derived pleural effusions using flow cytometry for a specific pattern of cell surface marker expression (CD44^+^, CD24^−^/low, and ESA^+^) (116–118). Many groups have attempted to confirm that the minimum surface phenotype for a tumorigenic BrCSC is CD44^+^CD24^−^ (119). In addition, CD133 (also a marker of CSC population in other tumors) and in some cases, selected members of the integrin family of receptors (beta1, alpha6, or beta3 integrins), alone or in conjunction with the CD44^+^/CD24^−^ phenotype have also been used to isolate the BrCSCs (120). Aldehyde dehydrogenase (ALDH) expression has similarly been used as a marker for BrCSCs (121). While these markers have exciting implications, it remains to be seen whether a single cell isolated by this method can develop new tumors in animal models.

### Breast cancer and DNMTs

In cancer, DNMTs are overexpressed in various tumor types, including breast cancer (28, 52, 122). Surprisingly, the mean levels of DNMT1, DNMT3a, and DNMT3b overexpression have turned out to be quite similar among different tumor types. The DNMT3b gene has shown the highest range of expression (81.8 for DNMT3a compared with 16.6 and 14 for DNMT1 and DNMT3a, respectively). In breast cancer, ~30% of patients revealed overexpression of DNMT3b in the tumor tissue as compared to normal breast tissue. Taking only these overexpressing tumors into account, the DNMT3b expression change was 82-fold, thus being significantly higher (28). Interestingly, DNMT1 and DNMT3a were overexpressed in only 5 and 3% of breast carcinomas (52). Thus, DNMT3b plays the predominant role over DNMT3a and DNMT1 in breast tumorigenesis. This is consistent with a recent study in breast cancer cell lines, which demonstrated a strong correlation between total DNMT activity and overexpression of DNMT3b, but not with the expression of DNMT3a or DNMT1 (52).

### Role of DNMT’s in other cancers

One study with prostate cancers demonstrated that the activity of DNMT1, DNMT3a, and DNMT3b are twofold to threefold higher in cancer cell lines and cancer tissues, as compared with a benign prostate epithelium cell line and benign prostatic hyperplasia tissues (27). Similarly, in a study with kidney cancer, expression of DNMT1 was higher in the carcinoma tissue compared to the adjacent normal tissue (123). DNMT1 and DNMT3b were also observed to be up to threefold higher in ovarian cancer cells than in normal ovarian surface epithelial cells (124, 125). Similarly, in hepatocellular carcinomas, there is increased expression of DNMT1, DNMT3a, and DNMT3b and a progressive increase in the number of methylated genes from normal liver, chronic hepatitis/cirrhosis to hepatocellular carcinoma. Furthermore, increase in the DNMT3a and DNMT3b mRNA in the carcinoma tissues relative to their non-cancerous normal may be a predictor of poor survival (30). Moreover, Mutze et al. had shown that DNMT1 is a predictive biomarker and potential therapeutic target for chemotherapy in gastric cancer (126). Another study demonstrated that DNMT1 and DNMT3b are overexpressed in gliomas (48).

Similarly in acute myelogenous leukemias, DNMT1, DNMT3a, and DNMT3b levels were significantly upregulated when compared to control bone marrow cells. Although CML cells in the chronic phase did not show significant changes, cells in the acute phase also showed increased levels of the three DNMTs. These data suggest that upregulated DNMTs may contribute to the pathogenesis of leukemia by inducing aberrant regional hypermethylation (44).

### Lacunas in the DNMT’s research in cancer biology

DNMT2 or TRDMT1 and DNMT3L are yet to be studied in detail in the different cancers. DNMT2 was shown to methylate tRNA (15, 127, 128). DNMT2 levels were found to be significantly lower in colorectal and stomach cancers than in non-cancerous tissue (50). In addition, significant overexpression of DNMT3b and reduced expression of DNMT2 were observed in hepatocellular carcinomas compared with the corresponding non-cancerous liver tissues (49). DNMT2 utilizes a DNMT mechanism for RNA methylation (129). DNMT2 activity can be monitored by tRNA^Asp^ methylation analysis and has been identified as a promising candidate biomarker for azacytidine therapy (129, 130).

DNMT3L is essential for the establishment of maternal genomic imprints but lacks key methyltransferase motifs and is possibly a regulator of methylation rather than an enzyme that methylates DNA (128, 131). Gokul et al. revealed that significant DNA methylation differences within the promoter of DNMT3L. A regulator of *de novo* DNMTs, DNMT3a and DNMT3b, DNMT3L promoter was found to have lost DNA methylation to varying levels in 14 out of 15 cancer cervix samples that were analyzed. This study highlights the importance of DNA methylation profile at DNMT3L promoter as a promising biomarker for cervical cancer and provides insight into the possible role of DNMT3L in cancer development (55). Another study demonstrated that DNMT3L is a novel marker and was essential for the growth of human embryonal carcinoma (56). Additional studies are required for the precise role for DNMT3L on other cancers.

### DNMTs promote cancer

Three DNMTs encoded by different genes are known to participate in generating and maintaining the DNA methylation pattern: DNMT1 (132), DNMT3a, and DNMT3b (133). Several studies have shown that DNMT1 is particularly involved in controlling cell growth (134, 135). Chik and Szyf determined whether specific inhibition of DNMT1 would increase the selectivity toward inhibition of cellular transformation and reduce the risk of increasing cell invasiveness. They found that depletion of DNMT1 had the strongest effect on colony growth suppression in cellular transformation but did not induce cellular invasion in MCF-7 and ZR-75-1 non-invasive breast cancer cell lines (136). Studies have also shown that overexpression of DNMT1 in non-transformed cells leads to cellular transformation (34), whereas knockout of DNMT1 protects mice from colorectal cancer (80). In addition, targeted deregulation of DNMTs by hepatitis B virus X protein promotes both specific regional hypermethylation and global hypomethylation. These epigenetic modulations by hepatitis B virus X protein may suggest a mechanism for epigenetic tumorigenesis during HBV-mediated hepatocarcinogenesis (137). Moreover, recent study demonstrated that interleukin (IL)-23, was shown to induce DNMT1 in a STAT5-dependent manner (138).

### Inhibition of DNMTs for anti-cancer therapy

Inhibition of DNMTs correlates with reduction in tumorigenicity and increased expression of tumor suppressor genes (139). Hence, DNMTs are considered valuable targets for the design of specific anti-cancer strategies. The three most commonly used catalytic inhibitors of DNMTs are the nucleoside analogs 5-azaC, 5-azaCdR, and zebularine. The mechanism of action of these three inhibitors is unique. These agents are pro-drugs that need to be incorporated into DNA to act as inhibitors of DNMTs. The nucleoside analogs are first phosphorylated to the triphosphate nucleotide and incorporated into DNA during DNA synthesis. DNMT1 forms a covalent bond with the carbon at position 6 of the cytosine as well as 5-aza-cytosine ring. Under normal conditions the enzyme transfers the methyl group from SAM to the fifth carbon position of the cytosine ring. This enables the release of the enzyme from its covalent bond with cytosine. When a 5′-aza-cytosine ring replaces cytosine in the DNA, the methyl transfer does not take place and the DNMT is trapped on the DNA (140). The replication fork progresses in the absence of DNMT1 resulting in passive loss of DNA methylation in the nascent strand but not the template. Zebularine is a nucleoside analog, which unlike 5-azaC is chemically stable and is orally bioavailable. Zebularine has been originally identified as a cytidine deaminase inhibitor (128, 141). Decitabine and 5-azacytidine are two well-known inhibitors of DNMTs that are effective against bone marrow disorders such as myelodysplastic syndrome (142). There are several DNMTs inhibitors that were studied in several cancers and these are summarized in Table 2.

**Table 2:** **Available DNMT in inhibitors and cancer**.

DNMT inhibitors	Dose range	Clinical trials	Reference
5-Azacytine	μM	Phase I, II, III: hematological malignancies	Kaminskas et al. (143), Yoo and Jones (144)
5-Aza-2′-deoxycytidine	μM	Phase I, II, III: hematological malignancies; cervical, non-small-cell lung cancer	Yoo and Jones (144), Prakash et al. (145), Pauer et al. (146)
5-Fluoro-2′-deoxycytidine	μM	Phase I	Yoo and Jones (144), Eidinoff and Rich (147)
5,6-Dihydro-5-azacytidine	μM	Phase I, II: ovarian cancer and lymphomas	Yoo and Jones (144), Curt et al. (148)
Zebularine	μM–mM		Cheng et al. (141), Yoo and Jones (144), Holleran et al. (149)
Hydralazine		Phase I: cervical cancer	Yoo and Jones (144), Zambrano et al. (150)
Procainamide		Preclinical	Yoo and Jones (144), Chuang et al. (151)
EGCG		Preclinical	Yoo and Jones (144), Fang et al. (152)
RG108		Preclinical	Yoo and Jones (144), Brueckner et al. (153)
SGI-110		Phase II: AML	Gros et al. (154)

## Chemopreventive Agents and DNMTs

**Figure 2:**
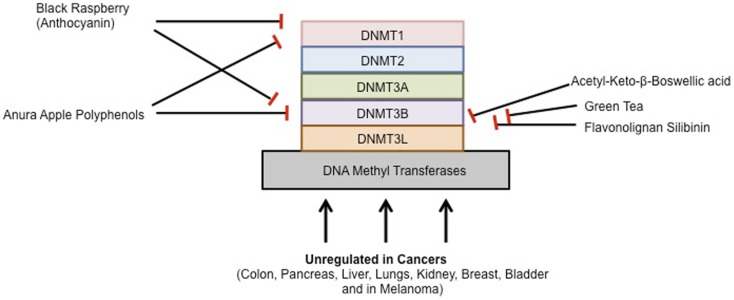
**Chemopreventive agents and DNMTs**.

To prevent, the onset of cancer, the National Institutes of Health (NIH) in the United States recommended a high fiber, low fat diet, consisting of more fruits and vegetables. Epidemiological studies suggest that diet plays a major role in the prevention of many cancers. The inhibitory effect of chemopreventive compounds on various DNMTs is shown in Figure 2.

Black Raspberry-derived anthocyanins were first shown to inhibit DNMT1 and DNMT3B in colon cancer cells. Oral administration of black raspberry powder (BRP) decreased promoter methylation of tumor suppressor genes in tumors from patients with colorectal cancer. The anthocyanins in black raspberries are responsible, at least in part, for their cancer-inhibitory effects. Three days of treatment with anthocyanins suppressed both expression and activity of DNMT1 and DNMT3B proteins in HCT116, Caco2, and SW480 cells. Promoters of CDKN2A, and SFRP2, SFRP5, and of WIF1, an inhibitor of the Wnt pathway, were demethylated by anthocyanins. Moreover, expression of β-catenin and c-Myc mRNA, downstream of Wnt pathway were decreased resulting in reduced cell proliferation and increased apoptosis. Anthocyanins were taken up into HCT116 cells and were differentially localized with DNMT1 and DNMT3B in the same cells visualized using confocal laser scanning microscopy. Although a previous study reported that DNMT3B is regulated by c-Myc in mouse lymphoma, DNMT3B did not bind with c-Myc in HCT116 cells. These findings suggest that anthocyanins are responsible, at least in part, for the demethylation effects of whole black raspberries in colorectal cancers (155). In another study, the biopsies of adjacent normal tissues and colorectal adenocarcinomas were taken from 20 patients before and after oral consumption of BRP (60 g/day) for 1–9 weeks. Methylation status of promoter regions of five tumor suppressor genes was quantified. Protein expression of DNMT1 and genes associated with cell proliferation, apoptosis, angiogenesis, and Wnt signaling were measured. They found that the methylation of three Wnt pathway inhibitors, SFRP2, SFRP5, and WIF1, and PAX6a, a developmental regulator, was modulated in a protective direction by BRBs in normal tissues and in colorectal tumors. Moreover, this effect was only observed in patients who received BRB treatment for an average of 4 weeks, but not in the 20 patients who received treatment for 1–9 weeks. This was associated with decreased expression of DNMT1. BRBs modulated expression of genes associated with Wnt pathway, proliferation, apoptosis, and angiogenesis in a protective direction. Their data provide evidence of the ability of BRBs to demethylate tumor suppressor genes and to modulate other biomarkers of tumor development in the human colon and rectum (156).

Annurca apple is an apple variety from southern Italy that is rich in polyphenols. Annurca apple polyphenol extracts inhibited the expression of DNMT1 and DNMT3b in colon cancer cells (157). Furthermore, a recent study demonstrated that acetyl-keto-β-boswellic acid, derived from the plant *Boswellia serrata*, an Indian frankincense inhibits DNMT activity in colorectal cancer cell lines (158). Most recently, the flavonolignan silibinin, which is the main pharmacologically active component of the milk thistle plant (*Silybum marianum*) with anti-cancer properties is able to significantly inhibit DNMT activity in colon cancer cells (159).

Green tea has also been shown to modulate DNMT activity to inhibit tumorigenesis. EGCG, the major polyphenol in green tea, has many interesting activities and is believed to be a key active ingredient. It dose-dependently inhibited DNMT activity, showing competitive inhibition with a Ki of 6.89 μM in human esophageal squamous cell carcinoma cell lines (152, 160). Moreover, a breast cancer study demonstrated that tea polyphenols [catechin, epicatechin, and (−)-epigallocatechin-3-*O*-gallate (EGCG)] and bioflavonoids (quercetin, fisetin, and myricetin) inhibited DNMT1-mediated DNA methylation in a dose dependent manner. The IC50 values for catechin, epicatechin, and various flavonoids ranged from 1.0 to 8.4 μM, but EGCG was a more potent inhibitor, with IC50 values ranging from 0.21 to 0.47 μM in MCF-7 and MDA-MB-231 cell lines (161, 162). Similarly, genistein present in soybean modulates enzymes that regulate DNA methylation and reactivates tumor suppressor genes in esophageal cancer cells (163, 164). Furthermore, two common catechol-containing coffee polyphenols such as caffeic acid and chlorogenic acid inhibits DNMT1 levels in breast cancer cells. The IC50 values of caffeic acid and chlorogenic acid were 3.0 and 0.75 μM, respectively (161). Curcumin, the active ingredient in turmeric has also been shown to affect DNMT activity. Virtual screening using a DNMT1 homology model suggested two binding modes for curcumin in the catalytic domain. Experimental inhibition of the bacterial C5 DNMT M. SssI confirmed that curcumin and derivatives can inhibit the protein with IC50 values around 30 nM. Curcumin at doses higher than 3 mM also induced a decrease of global DNA demethylation of leukemia MV4-11 cells (154, 165). Furthermore, a recent study demonstrated that curcumin down-regulates DNMT1 expression in AML cell lines, both *in vitro* and *in vivo*, and in primary AML cells *ex vivo* (166).

*Thymus serpyllum* (wild thyme) is an aromatic medicinal plant possessing several biological properties including anti-cancer activity. *T. serpyllum* extract induced significant cytotoxicity in breast cancer cells (MCF-7 and MDA-MB-231) but not in normal cells. It also induced apoptosis and inhibited the DNMT and HDAC activities in MDA-MB-231 cells (167). Recently, an identification of Kazinol Q, a natural product from formosan plants, was found to act as an inhibitor of DNMTs (168). In addition, a more recent study demonstrated that natural compounds such as EGCG, genistein, withaferin A, curcumin, resveratrol, and guggulsterone inhibit DNMT1, DNMT3a, and DNMT3b expression in breast cancer cell lines (169).

## Conclusion

The cancer stem cell hypothesis is gaining acceptance after the accumulation of extensive research evidence suggesting that the small subset of the tumor mass is responsible for the sustained growth of the tumor. Furthermore, it is becoming apparent that the cancer stem cells are responsible for disease relapse and resistance to the existing therapies. DMNTs have also upregulated in various cancers including colon, pancreatic, and breast cancers and their stem cells. Current DNMT inhibitors such as Decitabine and 5-azacytidine have adverse side effects. Identifying new drugs that can specifically target DNMTs and also cancer stem cells could lead to a new generation of anti-cancer medicines and with it, a new strategy for treatment. For instance, dietary phytochemicals are natural products found in our diet and can be used to target cancer stem cells. Thus, identification of such cancer stem cell targeting therapy and their use in combination with standard DNMT inhibitors will curtail this dreadful disease.

## Conflict of Interest Statement

The authors declare that the research was conducted in the absence of any commercial or financial relationships that could be construed as a potential conflict of interest.

## References

[1] Sato N Parker AR Fukushima N Miyagi Y Iacobuzio-Donahue CA Eshleman JR Epigenetic inactivation of TFPI-2 as a common mechanism associated with growth and invasion of pancreatic ductal adenocarcinoma. Oncogene (2005) 24(5):850–8 10.1038/sj.onc.1208050 15592528

[2] Kurdistani SK Histone modifications as markers of cancer prognosis: a cellular view. Br J Cancer (2007) 97(1):1–5 10.1038/sj.bjc.6603844 17592497 PMC2359665

[3] Esteller M Epigenetics in cancer. N Engl J Med (2008) 358(11):1148–59 10.1056/NEJMra072067 18337604

[4] Ghoshal K Bai S DNA methyltransferases as targets for cancer therapy. Drugs Today (Barc) (2007) 43(6):395–422 10.1358/dot.2007.43.6.1062666 17612710

[5] Robertson KD DNA methylation, methyltransferases, and cancer. Oncogene (2001) 20(24):3139–55 10.1038/sj.onc.1204341 11420731

[6] Ren J Singh BN Huang Q Li Z Gao Y Mishra P DNA hypermethylation as a chemotherapy target. Cell Signal (2011) 23(7):1082–93 10.1016/j.cellsig.2011.02.003 21345368

[7] Jin B Li Y Robertson KD DNA methylation: superior or subordinate in the epigenetic hierarchy? Genes Cancer (2011) 2(6):607–17 10.1177/1947601910393957 21941617 PMC3174260

[8] Miremadi A Oestergaard MZ Pharoah PD Caldas C Cancer genetics of epigenetic genes. Hum Mol Genet (2007) 16:R28–49 10.1093/hmg/ddm021 17613546

[9] Ferguson-Smith AC Greally JM Epigenetics: perceptive enzymes. Nature (2007) 449(7159):148–9 10.1038/449148a 17851501

[10] Okano M Xie S Li E Cloning and characterization of a family of novel mammalian DNA (cytosine-5) methyltransferases. Nat Genet (1998) 19(3):219–20 10.1038/890 9662389

[11] Malik K Brown KW Epigenetic gene deregulation in cancer. Br J Cancer (2000) 83(12):1583–8 10.1054/bjoc.2000.1549 11104550 PMC2363469

[12] Probst AV Dunleavy E Almouzni G Epigenetic inheritance during the cell cycle. Nat Rev Mol Cell Biol (2009) 10(3):192–206 10.1038/nrm2640 19234478

[13] Li E Bestor TH Jaenisch R Targeted mutation of the DNA methyltransferase gene results in embryonic lethality. Cell (1992) 69(6):915–26 10.1016/0092-8674(92)90611-F 1606615

[14] Hermann A Schmitt S Jeltsch A The human Dnmt2 has residual DNA-(cytosine-C5) methyltransferase activity. J Biol Chem (2003) 278(34):31717–21 10.1074/jbc.M305448200 12794065

[15] Goll MG Kirpekar F Maggert KA Yoder JA Hsieh CL Zhang X Methylation of tRNAAsp by the DNA methyltransferase homolog Dnmt2. Science (2006) 311(5759):395–8 10.1126/science.1120976 16424344

[16] Okano M Takebayashi S Okumura K Li E Assignment of cytosine-5 DNA methyltransferases Dnmt3a and Dnmt3b to mouse chromosome bands 12A2-A3 and 2H1 by in situ hybridization. Cytogenet Cell Genet (1999) 86(3–4):333–4 10.1159/000015331 10575238

[17] Xu GL Bestor TH Bourc’his D Hsieh CL Tommerup N Bugge M Chromosome instability and immunodeficiency syndrome caused by mutations in a DNA methyltransferase gene. Nature (1999) 402(6758):187–91 10.1038/46052 10647011

[18] Di Croce L Raker VA Corsaro M Fazi F Fanelli M Faretta M Methyltransferase recruitment and DNA hypermethylation of target promoters by an oncogenic transcription factor. Science (2002) 295(5557):1079–82 10.1126/science.1065173 11834837

[19] Kareta MS Botello ZM Ennis JJ Chou C Chedin F Reconstitution and mechanism of the stimulation of de novo methylation by human DNMT3L. J Biol Chem (2006) 281(36):25893–902 10.1074/jbc.M603140200 16829525

[20] Bourc’his D Viegas-Pequignot E Direct analysis of chromosome methylation. Methods Mol Biol (2001) 181:229–42 12843454 10.1385/1-59259-211-2:229

[21] Egger G Jeong S Escobar SG Cortez CC Li TW Saito Y Identification of DNMT1 (DNA methyltransferase 1) hypomorphs in somatic knockouts suggests an essential role for DNMT1 in cell survival. Proc Natl Acad Sci USA (2006) 103(38):14080–5 10.1073/pnas.0604602103 16963560 PMC1599915

[22] Li H Rauch T Chen ZX Szabo PE Riggs AD Pfeifer GP The histone methyltransferase SETDB1 and the DNA methyltransferase DNMT3A interact directly and localize to promoters silenced in cancer cells. J Biol Chem (2006) 281(28):19489–500 10.1074/jbc.M513249200 16682412

[23] Deplus R Brenner C Burgers WA Putmans P Kouzarides T de Launoit Y Dnmt3L is a transcriptional repressor that recruits histone deacetylase. Nucleic Acids Res (2002) 30(17):3831–8 10.1093/nar/gkf509 12202768 PMC137431

[24] Shi H Maier S Nimmrich I Yan PS Caldwell CW Olek A Oligonucleotide-based microarray for DNA methylation analysis: principles and applications. J Cell Biochem (2003) 88(1):138–43 10.1002/jcb.10313 12461783

[25] Robertson KD Uzvolgyi E Liang G Talmadge C Sumegi J Gonzales FA The human DNA methyltransferases (DNMTs) 1, 3a and 3b: coordinate mRNA expression in normal tissues and overexpression in tumors. Nucleic Acids Res (1999) 27(11):2291–8 10.1093/nar/27.11.2291 10325416 PMC148793

[26] el-Deiry WS Nelkin BD Celano P Yen RW Falco JP Hamilton SR High expression of the DNA methyltransferase gene characterizes human neoplastic cells and progression stages of colon cancer. Proc Natl Acad Sci USA (1991) 88(8):3470–4 10.1073/pnas.88.8.3470 2014266 PMC51469

[27] Patra SK Patra A Zhao H Dahiya R DNA methyltransferase and demethylase in human prostate cancer. Mol Carcinog (2002) 33(3):163–71 10.1002/mc.10033 11870882

[28] Girault I Tozlu S Lidereau R Bieche I Expression analysis of DNA methyltransferases 1, 3A, and 3B in sporadic breast carcinomas. Clin Cancer Res (2003) 9(12):4415–22 14555514

[29] Girault I Lerebours F Amarir S Tozlu S Tubiana-Hulin M Lidereau R Expression analysis of estrogen receptor alpha coregulators in breast carcinoma: evidence that NCOR1 expression is predictive of the response to tamoxifen. Clin Cancer Res (2003) 9(4):1259–66 12684393

[30] Oh BK Kim H Park HJ Shim YH Choi J Park C DNA methyltransferase expression and DNA methylation in human hepatocellular carcinoma and their clinicopathological correlation. Int J Mol Med (2007) 20(1):65–73 10.3892/ijmm.20.1.65 17549390

[31] Melki JR Warnecke P Vincent PC Clark SJ Increased DNA methyltransferase expression in leukaemia. Leukemia (1998) 12(3):311–6 10.1038/sj.leu.2400932 9529124

[32] Belinsky SA Nikula KJ Baylin SB Issa JP Increased cytosine DNA-methyltransferase activity is target-cell-specific and an early event in lung cancer. Proc Natl Acad Sci USA (1996) 93(9):4045–50 10.1073/pnas.93.9.4045 8633014 PMC39484

[33] Baylin SB Herman JG Graff JR Vertino PM Issa JP Alterations in DNA methylation: a fundamental aspect of neoplasia. Adv Cancer Res (1998) 72:141–96 10.1016/S0065-230X(08)60702-2 9338076

[34] Wu J Issa JP Herman J Bassett DE Jr. Nelkin BD Baylin SB Expression of an exogenous eukaryotic DNA methyltransferase gene induces transformation of NIH 3T3 cells. Proc Natl Acad Sci USA (1993) 90(19):8891–5 10.1073/pnas.90.19.8891 8415627 PMC47466

[35] Vertino PM Yen RW Gao J Baylin SB De novo methylation of CpG island sequences in human fibroblasts overexpressing DNA (cytosine-5-)-methyltransferase. Mol Cell Biol (1996) 16(8):4555–65 8754856 10.1128/mcb.16.8.4555 PMC231454

[36] Bakin AV Curran T Role of DNA 5-methylcytosine transferase in cell transformation by fos. Science (1999) 283(5400):387–90 10.1126/science.283.5400.387 9888853

[37] Laird PW Jaenisch R The role of DNA methylation in cancer genetic and epigenetics. Annu Rev Genet (1996) 30:441–64 10.1146/annurev.genet.30.1.441 8982461

[38] MacLeod AR Szyf M Expression of antisense to DNA methyltransferase mRNA induces DNA demethylation and inhibits tumorigenesis. J Biol Chem (1995) 270(14):8037–43 10.1074/jbc.270.14.8037 7713905

[39] MacLeod AR Rouleau J Szyf M Regulation of DNA methylation by the Ras signaling pathway. J Biol Chem (1995) 270(19):11327–37 10.1074/jbc.270.4.1595 7744770

[40] Ramchandani S MacLeod AR Pinard M von Hofe E Szyf M Inhibition of tumorigenesis by a cytosine-DNA, methyltransferase, antisense oligodeoxynucleotide. Proc Natl Acad Sci USA (1997) 94(2):684–9 10.1073/pnas.94.2.684 9012845 PMC19574

[41] Rountree MR Bachman KE Herman JG Baylin SB DNA methylation, chromatin inheritance, and cancer. Oncogene (2001) 20(24):3156–65 10.1038/sj.onc.1204339 11420732

[42] Rhee I Jair KW Yen RW Lengauer C Herman JG Kinzler KW CpG methylation is maintained in human cancer cells lacking DNMT1. Nature (2000) 404(6781):1003–7 10.1038/35010000 10801130

[43] Xie S Wang Z Okano M Nogami M Li Y He WW Cloning, expression and chromosome locations of the human DNMT3 gene family. Gene (1999) 236(1):87–95 10.1016/S0378-1119(99)00252-8 10433969

[44] Mizuno S Chijiwa T Okamura T Akashi K Fukumaki Y Niho Y Expression of DNA methyltransferases DNMT1, 3A, and 3B in normal hematopoiesis and in acute and chronic myelogenous leukemia. Blood (2001) 97(5):1172–9 10.1182/blood.V97.5.1172 11222358

[45] Yang J Wei X Wu Q Xu Z Gu D Jin Y Clinical significance of the expression of DNA methyltransferase proteins in gastric cancer. Mol Med Rep (2011) 4(6):1139–43 10.3892/mmr.2011.578 21887466

[46] Nagai M Nakamura A Makino R Mitamura K Expression of DNA (5-cytosin)-methyltransferases (DNMTs) in hepatocellular carcinomas. Hepatol Res (2003) 26(3):186–91 10.1016/S1386-6346(03)00091-3 12850690

[47] He S Wang F Yang L Guo C Wan R Ke A Expression of DNMT1 and DNMT3a are regulated by GLI1 in human pancreatic cancer. PLoS One (2011) 6(11):e27684 10.1371/journal.pone.0027684 22110720 PMC3215738

[48] Rajendran G Shanmuganandam K Bendre A Muzumdar D Goel A Shiras A Epigenetic regulation of DNA methyltransferases: DNMT1 and DNMT3B in gliomas. J Neurooncol (2011) 104(2):483–94 10.1007/s11060-010-0520-2 21229291

[49] Saito Y Kanai Y Sakamoto M Saito H Ishii H Hirohashi S Expression of mRNA for DNA methyltransferases and methyl-CpG-binding proteins and DNA methylation status on CpG islands and pericentromeric satellite regions during human hepatocarcinogenesis. Hepatology (2001) 33(3):561–8 10.1053/jhep.2001.22507 11230735

[50] Kanai Y Ushijima S Kondo Y Nakanishi Y Hirohashi S DNA methyltransferase expression and DNA methylation of CPG islands and peri-centromeric satellite regions in human colorectal and stomach cancers. Int J Cancer (2001) 91(2):205–12 10.1002/1097-0215(200002)9999:9999<::AID-IJC1040>3.0.CO;2-2 11146446

[51] Ley TJ Ding L Walter MJ McLellan MD Lamprecht T Larson DE DNMT3A mutations in acute myeloid leukemia. N Engl J Med (2010) 363(25):2424–33 10.1056/NEJMoa1005143 21067377 PMC3201818

[52] Roll JD Rivenbark AG Jones WD Coleman WB DNMT3b overexpression contributes to a hypermethylator phenotype in human breast cancer cell lines. Mol Cancer (2008) 7:15 10.1186/1476-4598-7-15 18221536 PMC2246151

[53] Ibrahim AE Arends MJ Silva AL Wyllie AH Greger L Ito Y Sequential DNA methylation changes are associated with DNMT3B overexpression in colorectal neoplastic progression. Gut (2011) 60(4):499–508 10.1136/gut.2010.223602 21068132

[54] Kobayashi Y Absher DM Gulzar ZG Young SR McKenney JK Peehl DM DNA methylation profiling reveals novel biomarkers and important roles for DNA methyltransferases in prostate cancer. Genome Res (2011) 21(7):1017–27 10.1101/gr.119487.110 21521786 PMC3129245

[55] Gokul G Gautami B Malathi S Sowjanya AP Poli UR Jain M DNA methylation profile at the DNMT3L promoter: a potential biomarker for cervical cancer. Epigenetics (2007) 2(2):80–5 10.4161/epi.2.2.3692 17965599 PMC2080824

[56] Minami K Chano T Kawakami T Ushida H Kushima R Okabe H DNMT3L is a novel marker and is essential for the growth of human embryonal carcinoma. Clin Cancer Res (2010) 16(10):2751–9 10.1158/1078-0432.CCR-09-3338 20460473

[57] Wilkinson N Scott-Conner CE Surgical therapy for colorectal adenocarcinoma. Gastroenterol Clin North Am (2008) 37(1):253–67 10.1016/j.gtc.2007.12.012 18313549

[58] Naishadham D Lansdorp-Vogelaar I Siegel R Cokkinides V Jemal A State disparities in colorectal cancer mortality patterns in the United States. Cancer Epidemiol Biomarkers Prev (2011) 20(7):1296–302 10.1158/1055-9965.EPI-11-0250 21737410

[59] Jemal A Siegel R Ward E Hao Y Xu J Murray T Cancer statistics, 2008. CA Cancer J Clin (2008) 58(2):71–96 10.3322/CA.2007.0010 18287387

[60] Siegel R Naishadham D Jemal A Cancer statistics, 2013. CA Cancer J Clin (2013) 63(1):11–30 10.3322/caac.21166 23335087

[61] DeSantis C Naishadham D Jemal A Cancer statistics for African Americans, 2013. CA Cancer J Clin (2013) 63(3):151–66 10.3322/caac.21173 23386565

[62] Siegel R Ma J Zou Z Jemal A Cancer statistics, 2014. CA Cancer J Clin (2014) 64(1):9–29 10.3322/caac.21208 24399786

[63] Young WF McGloin J Zittleman L West DR Westfall JM Predictors of colorectal screening in rural Colorado: testing to prevent colon cancer in the high plains research network. J Rural Health (2007) 23(3):238–45 10.1111/j.1748-0361.2007.00096.x 17565524

[64] Watt FM Driskell RR The therapeutic potential of stem cells. Philos Trans R Soc Lond B Biol Sci (2010) 365(1537):155–63 10.1098/rstb.2009.0149 20008393 PMC2842697

[65] Carpentino JE Hynes MJ Appelman HD Zheng T Steindler DA Scott EW Aldehyde dehydrogenase-expressing colon stem cells contribute to tumorigenesis in the transition from colitis to cancer. Cancer Res (2009) 69(20):8208–15 10.1158/0008-5472.CAN-09-1132 19808966 PMC2776663

[66] Diehn M Clarke MF Cancer stem cells and radiotherapy: new insights into tumor radioresistance. J Natl Cancer Inst (2006) 98(24):1755–7 10.1093/jnci/djj505 17179471

[67] Clarke MF Dick JE Dirks PB Eaves CJ Jamieson CH Jones DL Cancer stem cells – perspectives on current status and future directions: AACR workshop on cancer stem cells. Cancer Res (2006) 66(19):9339–44 10.1158/0008-5472.CAN-06-3126 16990346

[68] Dalerba P Clarke MF Cancer stem cells and tumor metastasis: first steps into uncharted territory. Cell Stem Cell (2007) 1(3):241–2 10.1016/j.stem.2007.08.012 18371356

[69] Huang EH Hynes MJ Zhang T Ginestier C Dontu G Appelman H Aldehyde dehydrogenase 1 is a marker for normal and malignant human colonic stem cells (SC) and tracks SC overpopulation during colon tumorigenesis. Cancer Res (2009) 69(8):3382–9 10.1158/0008-5472.CAN-08-4418 19336570 PMC2789401

[70] Huang EH Wicha MS Colon cancer stem cells: implications for prevention and therapy. Trends Mol Med (2008) 14(11):503–9 10.1016/j.molmed.2008.09.005 18929507 PMC2789402

[71] Ricci-Vitiani L Lombardi DG Pilozzi E Biffoni M Todaro M Peschle C Identification and expansion of human colon-cancer-initiating cells. Nature (2007) 445(7123):111–5 10.1038/nature05384 17122771

[72] Dean M Cancer stem cells: implications for cancer causation and therapy resistance. Discov Med (2005) 5(27):278–82 20704888

[73] Thenappan A Li Y Shetty K Johnson L Reddy EP Mishra L New therapeutics targeting colon cancer stem cells. Curr Colorectal Cancer Rep (2009) 5(4):209 10.1007/s11888-009-0029-2 20148131 PMC2818736

[74] Subramaniam D Ramalingam S Houchen CW Anant S Cancer stem cells: a novel paradigm for cancer prevention and treatment. Mini Rev Med Chem (2010) 10(5):359–71 10.2174/138955710791330954 20370703 PMC2874098

[75] Sureban SM May R Ramalingam S Subramaniam D Natarajan G Anant S Selective blockade of DCAMKL-1 results in tumor growth arrest by a Let-7a MicroRNA-dependent mechanism. Gastroenterology (2009) 137(2):649–59 10.1053/j.gastro.2009.05.004 19445940 PMC2775069

[76] May R Sureban SM Hoang N Riehl TE Lightfoot SA Ramanujam R Doublecortin and CaM kinase-like-1 and leucine-rich-repeat-containing G-protein-coupled receptor mark quiescent and cycling intestinal stem cells, respectively. Stem Cells (2009) 27(10):2571–9 10.1002/stem.193 19676123 PMC3049723

[77] Nakanishi Y Seno H Fukuoka A Ueo T Yamaga Y Maruno T Dclk1 distinguishes between tumor and normal stem cells in the intestine. Nat Genet (2013) 45(1):98–103 10.1038/ng.2481 23202126

[78] Metcalfe C de Sauvage FJ A tumor-specific stem cell. Nat Genet (2013) 45(1):7–9 10.1038/ng.2502 23268130

[79] Jin B Yao B Li JL Fields CR Delmas AL Liu C DNMT1 and DNMT3B modulate distinct polycomb-mediated histone modifications in colon cancer. Cancer Res (2009) 69(18):7412–21 10.1158/0008-5472.CAN-09-0116 19723660 PMC2745494

[80] Laird PW Jackson-Grusby L Fazeli A Dickinson SL Jung WE Li E Suppression of intestinal neoplasia by DNA hypomethylation. Cell (1995) 81(2):197–205 10.1016/0092-8674(95)90329-1 7537636

[81] Moser AR Pitot HC Dove WF A dominant mutation that predisposes to multiple intestinal neoplasia in the mouse. Science (1990) 247(4940):322–4 10.1126/science.2296722 2296722

[82] Ichii S Horii A Nakatsuru S Furuyama J Utsunomiya J Nakamura Y Inactivation of both APC alleles in an early stage of colon adenomas in a patient with familial adenomatous polyposis (FAP). Hum Mol Genet (1992) 1(6):387–90 10.1093/hmg/1.6.387 1338760

[83] Linhart HG Lin H Yamada Y Moran E Steine EJ Gokhale S Dnmt3b promotes tumorigenesis in vivo by gene-specific de novo methylation and transcriptional silencing. Genes Dev (2007) 21(23):3110–22 10.1101/gad.1594007 18056424 PMC2081977

[84] Eads CA Nickel AE Laird PW Complete genetic suppression of polyp formation and reduction of CpG-island hypermethylation in Apc(Min/+) Dnmt1-hypomorphic Mice. Cancer Res (2002) 62(5):1296–9 11888894

[85] Trinh BN Long TI Nickel AE Shibata D Laird PW DNA methyltransferase deficiency modifies cancer susceptibility in mice lacking DNA mismatch repair. Mol Cell Biol (2002) 22(9):2906–17 10.1128/MCB.22.9.2906-2917.2002 11940649 PMC133764

[86] Oghamian S Sodir NM Bashir MU Shen H Cullins AE Carroll CA Reduction of pancreatic acinar cell tumor multiplicity in Dnmt1 hypomorphic mice. Carcinogenesis (2011) 32(6):829–35 10.1093/carcin/bgr039 21362628 PMC3106433

[87] Trowbridge JJ Snow JW Kim J Orkin SH DNA methyltransferase 1 is essential for and uniquely regulates hematopoietic stem and progenitor cells. Cell Stem Cell (2009) 5(4):442–9 10.1016/j.stem.2009.08.016 19796624 PMC2767228

[88] Broske AM Vockentanz L Kharazi S Huska MR Mancini E Scheller M DNA methylation protects hematopoietic stem cell multipotency from myeloerythroid restriction. Nat Genet (2009) 41(11):1207–15 10.1038/ng.463 19801979

[89] Sen GL Reuter JA Webster DE Zhu L Khavari PA DNMT1 maintains progenitor function in self-renewing somatic tissue. Nature (2010) 463(7280):563–7 10.1038/nature08683 20081831 PMC3050546

[90] Morita R Hirohashi Y Suzuki H Takahashi A Tamura Y Kanaseki T DNA methyltransferase 1 is essential for initiation of the colon cancers. Exp Mol Pathol (2013) 94(2):322–9 10.1016/j.yexmp.2012.10.004 23064049

[91] Inoue M Yamamoto S Kurahashi N Iwasaki M Sasazuki S Tsugane S Daily total physical activity level and total cancer risk in men and women: results from a large-scale population-based cohort study in Japan. Am J Epidemiol (2008) 168(4):391–403 10.1093/aje/kwn146 18599492

[92] Nieto J Grossbard ML Kozuch P Metastatic pancreatic cancer 2008: is the glass less empty? Oncologist (2008) 13(5):562–76 10.1634/theoncologist.2007-0181 18515741

[93] Duffy JP Reber HA Pancreatic neoplasms. Curr Opin Gastroenterol (2003) 19(5):458–66 10.1097/00001574-200309000-00004 15703589

[94] Petrelli F Borgonovo K Ghilardi M Cabiddu M Barni S What else in gemcitabine-pretreated advanced pancreatic cancer? An update of second line therapies. Rev Recent Clin Trials (2010) 5(1):43–56 10.2174/157488710790820553 20205687

[95] Koutsounas I Giaginis C Theocharis S Histone deacetylase inhibitors and pancreatic cancer: are there any promising clinical trials? World J Gastroenterol (2013) 19(8):1173–81 10.3748/wjg.v19.i8.1173 23482354 PMC3587473

[96] Koutsounas I Giaginis C Patsouris E Theocharis S Current evidence for histone deacetylase inhibitors in pancreatic cancer. World J Gastroenterol (2013) 19(6):813–28 10.3748/wjg.v19.i6.813 23430136 PMC3574878

[97] Kawasaki BT Hurt EM Mistree T Farrar WL Targeting cancer stem cells with phytochemicals. Mol Interv (2008) 8(4):174–84 10.1124/mi.8.4.9 18829843

[98] Lee CJ Li C Simeone DM Human pancreatic cancer stem cells: implications for how we treat pancreatic cancer. Transl Oncol (2008) 1(1):14–8 10.1593/tlo.08013 18607507 PMC2510763

[99] Rhim AD Mirek ET Aiello NM Maitra A Bailey JM McAllister F EMT and dissemination precede pancreatic tumor formation. Cell (2012) 148(1–2):349–61 10.1016/j.cell.2011.11.025 22265420 PMC3266542

[100] May R Sureban SM Lightfoot SA Hoskins AB Brackett DJ Postier RG Identification of a novel putative pancreatic stem/progenitor cell marker DCAMKL-1 in normal mouse pancreas. Am J Physiol Gastrointest Liver Physiol (2010) 299(2):G303–10 10.1152/ajpgi.00146.2010 20522640 PMC2928534

[101] Sureban SM May R Lightfoot SA Hoskins AB Lerner M Brackett DJ DCAMKL-1 regulates epithelial-mesenchymal transition in human pancreatic cells through a miR-200a-dependent mechanism. Cancer Res (2011) 71(6):2328–38 10.1158/0008-5472.CAN-10-2738 21285251 PMC3072762

[102] Sato N Fukushima N Hruban RH Goggins M CpG island methylation profile of pancreatic intraepithelial neoplasia. Mod Pathol (2008) 21(3):238–44 10.1038/modpathol.3800991 18157091 PMC2678810

[103] Jacks T Remington L Williams BO Schmitt EM Halachmi S Bronson RT Tumor spectrum analysis in p53-mutant mice. Curr Biol (1994) 4(1):1–7 10.1016/S0960-9822(00)00002-6 7922305

[104] Clarke AR Cummings MC Harrison DJ Interaction between murine germline mutations in p53 and APC predisposes to pancreatic neoplasia but not to increased intestinal malignancy. Oncogene (1995) 11(9):1913–20 7478622

[105] Clarke AR Murine models of neoplasia: functional analysis of the tumour suppressor genes Rb-1 and p53. Cancer Metastasis Rev (1995) 14(2):125–48 10.1007/BF00665796 7554030

[106] Peng DF Kanai Y Sawada M Ushijima S Hiraoka N Kosuge T Increased DNA methyltransferase 1 (DNMT1) protein expression in precancerous conditions and ductal carcinomas of the pancreas. Cancer Sci (2005) 96(7):403–8 10.1111/j.1349-7006.2005.00071.x 16053511 PMC11159105

[107] Peng DF Kanai Y Sawada M Ushijima S Hiraoka N Kitazawa S DNA methylation of multiple tumor-related genes in association with overexpression of DNA methyltransferase 1 (DNMT1) during multistage carcinogenesis of the pancreas. Carcinogenesis (2006) 27(6):1160–8 10.1093/carcin/bgi361 16537562

[108] Peng JC Karpen GH H3K9 methylation and RNA interference regulate nucleolar organization and repeated DNA stability. Nat Cell Biol (2007) 9(1):25–35 10.1038/ncb1514 17159999 PMC2819265

[109] Wang W Gao J Man XH Li ZS Gong YF Significance of DNA methyltransferase-1 and histone deacetylase-1 in pancreatic cancer. Oncol Rep (2009) 21(6):1439–47 10.3892/or_00000372 19424621

[110] Zhang JJ Zhu Y Zhu Y Wu JL Liang WB Zhu R Association of increased DNA methyltransferase expression with carcinogenesis and poor prognosis in pancreatic ductal adenocarcinoma. Clin Transl Oncol (2012) 14(2):116–24 10.1007/s12094-012-0770-x 22301400

[111] Jemal A Bray F Center MM Ferlay J Ward E Forman D Global cancer statistics. CA Cancer J Clin (2011) 61(2):69–90 10.3322/caac.20107 21296855

[112] DeSantis C Siegel R Bandi P Jemal A Breast cancer statistics, 2011. CA Cancer J Clin (2011) 61(6):409–18 10.3322/caac.20134 21969133

[113] Sotiriou C Neo SY McShane LM Korn EL Long PM Jazaeri A Breast cancer classification and prognosis based on gene expression profiles from a population-based study. Proc Natl Acad Sci USA (2003) 100(18):10393–8 10.1073/pnas.1732912100 12917485 PMC193572

[114] Sorlie T Perou CM Tibshirani R Aas T Geisler S Johnsen H Gene expression patterns of breast carcinomas distinguish tumor subclasses with clinical implications. Proc Natl Acad Sci USA (2001) 98(19):10869–74 10.1073/pnas.191367098 11553815 PMC58566

[115] Isakoff SJ Triple-negative breast cancer: role of specific chemotherapy agents. Cancer J (2010) 16(1):53–61 10.1097/PPO.0b013e3181d24ff7 20164691 PMC2882502

[116] Chang WW Lee CH Lee P Lin J Hsu CW Hung JT Expression of Globo H and SSEA3 in breast cancer stem cells and the involvement of fucosyl transferases 1 and 2 in Globo H synthesis. Proc Natl Acad Sci USA (2008) 105(33):11667–72 10.1073/pnas.0804979105 18685093 PMC2575305

[117] Velasco-Velazquez MA Homsi N De La Fuente M Pestell RG Breast cancer stem cells. Int J Biochem Cell Biol (2012) 44(4):573–7 10.1016/j.biocel.2011.12.020 22249027 PMC3294043

[118] Velasco-Velazquez MA Popov VM Lisanti MP Pestell RG The role of breast cancer stem cells in metastasis and therapeutic implications. Am J Pathol (2011) 179(1):2–11 10.1016/j.ajpath.2011.03.005 21640330 PMC3123864

[119] Al-Hajj M Wicha MS Benito-Hernandez A Morrison SJ Clarke MF Prospective identification of tumorigenic breast cancer cells. Proc Natl Acad Sci USA (2003) 100(7):3983–8 10.1073/pnas.0530291100 12629218 PMC153034

[120] Lawson JC Blatch GL Edkins AL Cancer stem cells in breast cancer and metastasis. Breast Cancer Res Treat (2009) 118(2):241–54 10.1007/s10549-009-0524-9 19731012

[121] Charafe-Jauffret E Ginestier C Iovino F Tarpin C Diebel M Esterni B Aldehyde dehydrogenase 1-positive cancer stem cells mediate metastasis and poor clinical outcome in inflammatory breast cancer. Clin Cancer Res (2010) 16(1):45–55 10.1158/1078-0432.CCR-09-1630 20028757 PMC2874875

[122] Veeck J Esteller M Breast cancer epigenetics: from DNA methylation to microRNAs. J Mammary Gland Biol Neoplasia (2010) 15(1):5–17 10.1007/s10911-010-9165-1 20101446 PMC2824126

[123] Arai E Kanai Y Ushijima S Fujimoto H Mukai K Hirohashi S Regional DNA hypermethylation and DNA methyltransferase (DNMT) 1 protein overexpression in both renal tumors and corresponding nontumorous renal tissues. Int J Cancer (2006) 119(2):288–96 10.1002/ijc.21807 16453286

[124] Ahluwalia A Hurteau JA Bigsby RM Nephew KP DNA methylation in ovarian cancer. II. Expression of DNA methyltransferases in ovarian cancer cell lines and normal ovarian epithelial cells. Gynecol Oncol (2001) 82(2):299–304 10.1006/gyno.2001.6284 11531283

[125] Chen CL Yan X Gao YN Liao QP [Expression of DNA methyltransferase 1, 3A and 3B mRNA in the epithelial ovarian carcinoma]. Zhonghua Fu Chan Ke Za Zhi (2005) 40(11):770–4 16324253

[126] Mutze K Langer R Schumacher F Becker K Ott K Novotny A DNA methyltransferase 1 as a predictive biomarker and potential therapeutic target for chemotherapy in gastric cancer. Eur J Cancer (2011) 47(12):1817–25 10.1016/j.ejca.2011.02.024 21458988

[127] Rai K Chidester S Zavala CV Manos EJ James SR Karpf AR Dnmt2 functions in the cytoplasm to promote liver, brain, and retina development in *zebrafish*. Genes Dev (2007) 21(3):261–6 10.1101/gad.1472907 17289917 PMC1785123

[128] Szyf M DNA methylation and demethylation probed by small molecules. Biochim Biophys Acta (2010) 1799(10–12):750–9 10.1016/j.bbagrm.2010.09.002 20840878

[129] Schaefer M Lyko F Solving the Dnmt2 enigma. Chromosoma (2010) 119(1):35–40 10.1007/s00412-009-0240-6 19730874

[130] Schaefer M Hagemann S Hanna K Lyko F Azacytidine inhibits RNA methylation at DNMT2 target sites in human cancer cell lines. Cancer Res (2009) 69(20):8127–32 10.1158/0008-5472.CAN-09-0458 19808971

[131] Bourc’his D Xu GL Lin CS Bollman B Bestor TH Dnmt3L and the establishment of maternal genomic imprints. Science (2001) 294(5551):2536–9 10.1126/science.1065848 11719692

[132] Bestor TH Cloning of a mammalian DNA methyltransferase. Gene (1988) 74(1):9–12 10.1016/0378-1119(88)90238-7 3248734

[133] Okano M Bell DW Haber DA Li E DNA methyltransferases Dnmt3a and Dnmt3b are essential for de novo methylation and mammalian development. Cell (1999) 99(3):247–57 10.1016/S0092-8674(00)81656-6 10555141

[134] Szyf M The role of DNA methyltransferase 1 in growth control. Front Biosci (2001) 6:D599–609 10.2741/szyf 11282571

[135] Szyf M Detich N Regulation of the DNA methylation machinery and its role in cellular transformation. Prog Nucleic Acid Res Mol Biol (2001) 69:47–79 10.1016/S0079-6603(01)69044-5 11550798

[136] Chik F Szyf M Effects of specific DNMT gene depletion on cancer cell transformation and breast cancer cell invasion; toward selective DNMT inhibitors. Carcinogenesis (2011) 32(2):224–32 10.1093/carcin/bgq221 20980350

[137] Park IY Sohn BH Yu E Suh DJ Chung YH Lee JH Aberrant epigenetic modifications in hepatocarcinogenesis induced by hepatitis B virus X protein. Gastroenterology (2007) 132(4):1476–94 10.1053/j.gastro.2007.01.034 17408664

[138] Zhang L Li J Li L Zhang J Wang X Yang C IL-23 selectively promotes the metastasis of colorectal carcinoma cells with impaired Socs3 expression via the STAT5 pathway. Carcinogenesis (2014). 10.1093/carcin/bgu017 24464786

[139] Suzuki T Miyata N Non-hydroxamate histone deacetylase inhibitors. Curr Med Chem (2005) 12(24):2867–80 10.2174/092986705774454706 16305476

[140] Wu JC Santi DV On the mechanism and inhibition of DNA cytosine methyltransferases. Prog Clin Biol Res (1985) 198:119–29 4070306

[141] Cheng JC Matsen CB Gonzales FA Ye W Greer S Marquez VE Inhibition of DNA methylation and reactivation of silenced genes by zebularine. J Natl Cancer Inst (2003) 95(5):399–409 10.1093/jnci/95.5.399 12618505

[142] Khan H Vale C Bhagat T Verma A Role of DNA methylation in the pathogenesis and treatment of myelodysplastic syndromes. Semin Hematol (2013) 50(1):16–37 10.1053/j.seminhematol.2013.01.001 23507481

[143] Kaminskas E Farrell AT Wang YC Sridhara R Pazdur R FDA drug approval summary: azacitidine (5-azacytidine, Vidaza) for injectable suspension. Oncologist (2005) 10(3):176–82 10.1634/theoncologist.10-3-176 15793220

[144] Yoo CB Jones PA Epigenetic therapy of cancer: past, present and future. Nat Rev Drug Discov (2006) 5(1):37–50 10.1038/nrd1930 16485345

[145] Prakash S Foster BJ Meyer M Wozniak A Heilbrun LK Flaherty L Chronic oral administration of CI-994: a phase 1 study. Invest New Drugs (2001) 19(1):1–11 10.1023/A:1006489328324 11291827

[146] Pauer LR Olivares J Cunningham C Williams A Grove W Kraker A Phase I study of oral CI-994 in combination with carboplatin and paclitaxel in the treatment of patients with advanced solid tumors. Cancer Invest (2004) 22(6):886–96 10.1081/CNV-200039852 15641487

[147] Eidinoff ML Rich MA Growth inhibition of a human tumor cell strain by 5-fluoro-2-deoxyuridine: time parameters for subsequent reversal by thymidine. Cancer Res (1959) 19(5):521–4 13663041

[148] Curt GA Kelley JA Fine RL Huguenin PN Roth JS Batist G A phase I and pharmacokinetic study of dihydro-5-azacytidine (NSC 264880). Cancer Res (1985) 45(7):3359–63 2408749

[149] Holleran JL Parise RA Joseph E Eiseman JL Covey JM Glaze ER Plasma pharmacokinetics, oral bioavailability, and interspecies scaling of the DNA methyltransferase inhibitor, zebularine. Clin Cancer Res (2005) 11(10):3862–8 10.1158/1078-0432.CCR-04-2406 15897587

[150] Zambrano P Segura-Pacheco B Perez-Cardenas E Cetina L Revilla-Vazquez A Taja-Chayeb L A phase I study of hydralazine to demethylate and reactivate the expression of tumor suppressor genes. BMC Cancer (2005) 5:44 10.1186/1471-2407-5-44 15862127 PMC1131894

[151] Chuang JC Yoo CB Kwan JM Li TW Liang G Yang AS Comparison of biological effects of non-nucleoside DNA methylation inhibitors versus 5-aza-2’-deoxycytidine. Mol Cancer Ther (2005) 4(10):1515–20 10.1158/1535-7163.MCT-05-0172 16227400

[152] Fang MZ Wang Y Ai N Hou Z Sun Y Lu H Tea polyphenol (−)-epigallocatechin-3-gallate inhibits DNA methyltransferase and reactivates methylation-silenced genes in cancer cell lines. Cancer Res (2003) 63(22):7563–70 14633667

[153] Brueckner B Garcia Boy R Siedlecki P Musch T Kliem HC Zielenkiewicz P Epigenetic reactivation of tumor suppressor genes by a novel small-molecule inhibitor of human DNA methyltransferases. Cancer Res (2005) 65(14):6305–11 10.1158/0008-5472.CAN-04-2957 16024632

[154] Gros C Fahy J Halby L Dufau I Erdmann A Gregoire JM DNA methylation inhibitors in cancer: recent and future approaches. Biochimie (2012) 94(11):2280–96 10.1016/j.biochi.2012.07.025 22967704

[155] Wang LS Kuo CT Cho SJ Seguin C Siddiqui J Stoner K Black raspberry-derived anthocyanins demethylate tumor suppressor genes through the inhibition of DNMT1 and DNMT3B in colon cancer cells. Nutr Cancer (2013) 65(1):118–25 10.1080/01635581.2013.741759 23368921 PMC3570951

[156] Wang LS Arnold M Huang YW Sardo C Seguin C Martin E Modulation of genetic and epigenetic biomarkers of colorectal cancer in humans by black raspberries: a phase I pilot study. Clin Cancer Res (2011) 17(3):598–610 10.1158/1078-0432.CCR-10-1260 21123457 PMC3076314

[157] Fini L Selgrad M Fogliano V Graziani G Romano M Hotchkiss E Annurca apple polyphenols have potent demethylating activity and can reactivate silenced tumor suppressor genes in colorectal cancer cells. J Nutr (2007) 137(12):2622–8 18029474 10.1093/jn/137.12.2622

[158] Shen Y Takahashi M Byun HM Link A Sharma N Balaguer F Boswellic acid induces epigenetic alterations by modulating DNA methylation in colorectal cancer cells. Cancer Biol Ther (2012) 13(7):542–52 10.4161/cbt.19604 22415137 PMC3364790

[159] Kauntz H Bousserouel S Gosse F Raul F Epigenetic effects of the natural flavonolignan silibinin on colon adenocarcinoma cells and their derived metastatic cells. Oncol Lett (2013) 5(4):1273–7 10.3892/ol.2013.1190 23599778 PMC3629096

[160] Gilbert ER Liu D Flavonoids influence epigenetic-modifying enzyme activity: structure – function relationships and the therapeutic potential for cancer. Curr Med Chem (2010) 17(17):1756–68 10.2174/092986710791111161 20345345

[161] Lee WJ Shim JY Zhu BT Mechanisms for the inhibition of DNA methyltransferases by tea catechins and bioflavonoids. Mol Pharmacol (2005) 68(4):1018–30 10.1124/mol.104.008367 16037419

[162] Li Y Tollefsbol TO Impact on DNA methylation in cancer prevention and therapy by bioactive dietary components. Curr Med Chem (2010) 17(20):2141–51 10.2174/092986710791299966 20423306 PMC2904405

[163] Fang MZ Jin Z Wang Y Liao J Yang GY Wang LD Promoter hypermethylation and inactivation of O(6)-methylguanine-DNA methyltransferase in esophageal squamous cell carcinomas and its reactivation in cell lines. Int J Oncol (2005) 26(3):615–22 10.3892/ijo.26.3.615 15703815

[164] Huang YW Kuo CT Stoner K Huang TH Wang LS An overview of epigenetics and chemoprevention. FEBS Lett (2011) 585(13):2129–36 10.1016/j.febslet.2010.11.002 21056563 PMC3071863

[165] Liu Z Xie Z Jones W Pavlovicz RE Liu S Yu J Curcumin is a potent DNA hypomethylation agent. Bioorg Med Chem Lett (2009) 19(3):706–9 10.1016/j.bmcl.2008.12.041 19112019

[166] Yu J Peng Y Wu LC Xie Z Deng Y Hughes T Curcumin down- regulates DNA methyltransferase 1 and plays an anti-leukemic role in acute myeloid leukemia. PLoS One (2013) 8(2):e55934 10.1371/journal.pone.0055934 23457487 PMC3572185

[167] Bozkurt E Atmaca H Kisim A Uzunoglu S Uslu R Karaca B Effects of *Thymus serpyllum* extract on cell proliferation, apoptosis and epigenetic events in human breast cancer cells. Nutr Cancer (2012) 64(8):1245–50 10.1080/01635581.2012.719658 23163852

[168] Weng JR Lai IL Yang HC Lin CN Bai LY Identification of Kazinol Q, a natural product from formosan plants, as an inhibitor of DNA methyltransferase. Phytother Res (2014) 28(1):49–54 10.1002/ptr.4955 23447335

[169] Mirza S Sharma G Parshad R Gupta SD Pandya P Ralhan R Expression of DNA methyltransferases in breast cancer patients and to analyze the effect of natural compounds on DNA methyltransferases and associated proteins. J Breast Cancer (2013) 16(1):23–31 10.4048/jbc.2013.16.1.23 23593078 PMC3625766

